# Climate Change and Crop Exposure to Adverse Weather: Changes to Frost Risk and Grapevine Flowering Conditions

**DOI:** 10.1371/journal.pone.0141218

**Published:** 2015-10-23

**Authors:** Jonathan R. Mosedale, Robert J. Wilson, Ilya M. D. Maclean

**Affiliations:** 1 Environment and Sustainability Institute, College of Life and Environmental Sciences, University of Exeter, Penryn Campus, Penryn, United Kingdom; 2 College of Life and Environmental Sciences, Hatherly Building, Prince of Wales Road, Exeter, United Kingdom; Universidade de Vigo, SPAIN

## Abstract

The cultivation of grapevines in the UK and many other cool climate regions is expected to benefit from the higher growing season temperatures predicted under future climate scenarios. Yet the effects of climate change on the risk of adverse weather conditions or events at key stages of crop development are not always captured by aggregated measures of seasonal or yearly climates, or by downscaling techniques that assume climate variability will remain unchanged under future scenarios. Using fine resolution projections of future climate scenarios for south-west England and grapevine phenology models we explore how risks to cool-climate vineyard harvests vary under future climate conditions. Results indicate that the risk of adverse conditions during flowering declines under all future climate scenarios. In contrast, the risk of late spring frosts increases under many future climate projections due to advancement in the timing of budbreak. Estimates of frost risk, however, were highly sensitive to the choice of phenology model, and future frost exposure declined when budbreak was calculated using models that included a winter chill requirement for dormancy break. The lack of robust phenological models is a major source of uncertainty concerning the impacts of future climate change on the development of cool-climate viticulture in historically marginal climatic regions.

## Introduction

To ensure future global and regional food security there is an urgent need to explore the implications of climate change not only on existing crop yields but also on the suitability for novel crop species [1, 2]. Climate modelling however does not provide precise or certain predictions of future conditions in agricultural regions, and therefore any adaptation must be based upon probabilistic projections that take into account the range of uncertainty in projections of future climate.

Due to the coarse spatial and temporal resolution of most climate models, attempts to assess the effect of climate change on agricultural crops have focussed on modelling how changes in mean climate parameters and metrics, such as yearly or seasonal mean temperatures and precipitation, affect yields. Yet climate change is not restricted to changes in mean conditions, but also involves changes to climate variability, seasonal weather patterns and the frequency and magnitude of extreme weather events [3, 4]. Importantly, the yields of many agricultural crops are not only affected by mean seasonal conditions, but are also vulnerable to the risk of damaging or unsuitable weather at key stages of crop development, which are not readily captured by metrics of yearly, seasonal or even monthly climate parameters, or by downscaling methods that assume historical measures of climate variability will prevail under future conditions [5]. As a result, we possess limited knowledge of how certain key agricultural risks will vary under future climate projections.

Stochastic weather generators [6] have been used for several years to downscale the outputs of future climate models, allowing the generation of daily weather simulations at relatively high spatial resolutions under different climate scenarios that are able to capture changes in climate variability and seasonal weather patterns. The availability and use of weather generator data has been enhanced by the creation of user interfaces such as the UKCP09 weather generator [7] and the Environment Agency Rainfall and Weather Impacts Generator [8] in the United Kingdom. Weather generators have become favoured analytical tools within key industry sectors such as the water industry [9], for whom the timing and sequence of weather events is of critical importance for assessing the implications of climate change for water resource provision and flood management. Stochastic weather generators are also used with crop simulation models to explore climate change impacts on agriculture [10]. The ability to generate multiple simulations of weather sequences allows calculation of the risks of weather events at key stages of crop development [11], and the daily resolution of weather sequences permits the use of phenological models of crop development that are commonly based on daily mean temperature accumulation [12].

The cultivation of grapevines for wine production, or viticulture, is expected to benefit from climate change projections for the United Kingdom [13, 14] and several other temperate regions [15–19]. The past 20 years have already seen considerable growth in UK vineyard acreage, wine sales and market reputation [20], while several other cool-climate viticulture regions have also shown high growth over recent decades [21, 22]. As a perennial fruit crop with a long productive life and high establishment costs, viticulture is an example of an agricultural sector that could greatly benefit from reliable information about future climate risks to inform decisions on vineyard location, cultivar choice and crop management.

The expectation of improved growing conditions in many cool-climate viticulture regions is predominantly based upon a projected increase in mean growing season temperatures, viewed as the main factor defining the upper latitude limits of viticulture [23] and restricting the range of suitable cultivars that can be grown [24, 25]. However, year-on-year vineyard yields are highly variable [26] and this is particularly the case for marginal regions such as the United Kingdom where the average national productivity has varied from under 4 to over 30 hectolitres per hectare over the past five years [20], and where the loss of whole annual harvests from established vineyards is a recognized phenomenon. Yields are susceptible to weather events and conditions at key phenological stages during the growing season, such as at budbreak and flowering [27]. Spring frost is a risk to viticulture in many cool-climate regions [28, 29] causing significant crop loss if it occurs after bud-burst [30, 31]. Among the many environmental factors that can affect flowering and fruit-set [32], cold or rainy weather around flowering can greatly reduce the number of grape clusters formed and thereby harvest yields [33, 34]. Late spring frosts and adverse weather at flowering are risks not only to cool-climate viticulture, but also to the quality and yields of many other crops [5, 35–38].

In this paper we demonstrate how stochastic weather generators can be used to quantify the effects of climate change on agricultural risks associated with weather events at key phases of crop development. Taking viticulture in south-west England as an example of a currently marginal crop predicted to benefit from climate change, we examine how key risks to vineyard yields, namely the likelihood of late spring frost after budbreak and the suitability of weather at flowering, vary under future climate projections. Importantly, we consider how climate change will affect both weather conditions at these key stages of development and the timing of developmental stages as warmer temperatures promote more advanced phenology [39–42].

## Materials and Methods

### Study site and identification of weather risks

**Fig 1:**
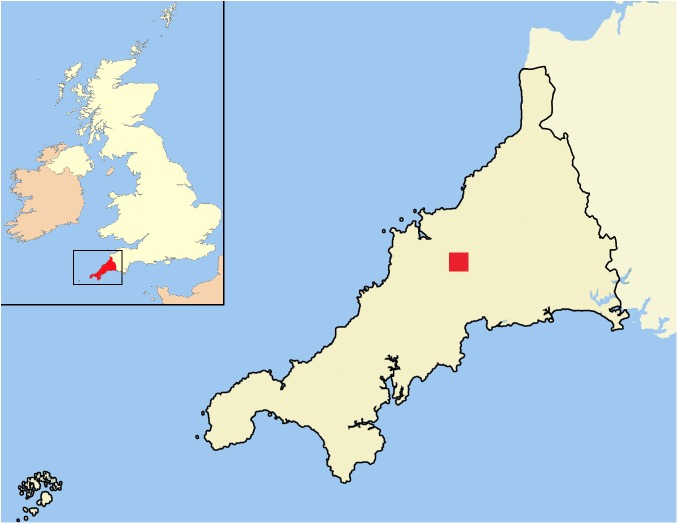
Location of 5x5 km study site within Cornwall in south-west England.

South-west England lies at the margins of UK viticulture, with no clearly defined grape-growing region but nonetheless home to several commercial vineyards and wineries extending down to the most westerly county of Cornwall (Fig 1). The Atlantic maritime climate is characterised by mild winters but lower temperatures and higher precipitation during the grapevine growing season than in south-east England, where the majority of UK viticulture is located.

A list of weather parameters cited as affecting the yields and quality of marginal, cool-climate vineyards was drawn up from consultation with the literature. These parameters were then used to inform semi-structured interviews, of up to 45 minutes, with seven vineyard owners or winemakers from Cornwall and south Devon who were contacted in advance and gave informed and written consent on agreement of anonymity. Interviewees were asked to identify those parameters they considered of most importance to their ‘success’–the term being chosen to cover both effects on yield and quality while recognizing that not all vineyards were viewed as self-sustaining economic concerns. The subject’s participation was voluntary and the research methods received approval from University of Exeter Bioscience—Ethics committee approval (2014/648).

All of those interviewed identified rainy or cold weather at flowering, spring frosts after budburst and low summer temperatures as the three most important risks affecting their success.

To explore how these risks are likely to vary under different climate scenarios, involves three steps: (i) the collation of historic weather records and the generation of daily weather sequences under future climate scenarios for an established vineyard location, (ii) the selection of appropriate phenological models to calculate the timing of flowering and budbreak, which together allow (iii) the definition and calculation of risk exposure. Each of these steps is described in the following sections.

### Climate modelling and weather sequence generation

Future climate projections used the latest UK Climate Impacts Programme climatology, termed UKCP09 [43], which adopts a Bayesian framework to calculate probability distributions of climate responses to increased CO_2_ emissions. Projections derive from a perturbed parameter model ensemble based on the HadCM3 model [44, 45]; also incorporating multimodel ensembles of other international climate models and adjusted according to how well simulations fit historical climate observations. Downscaling using an ensemble of RCM variants configured from the HadCM3 model give probability distribution functions at a resolution of 25 km—each distribution function the product of 10 000 equiprobable “possibilities” describing an internally consistent set of climate variables.

Further stochastic downscaling is provided by the UKCP09 weather generator to produce statistically credible representations of daily weather patterns to a resolution of 5 x 5 km. The weather generator uses a stochastic precipitation model [8] to generate daily precipitation patterns which are then used by a second stochastic model to generate other weather variables conditioned on the rainfall series, resulting in an internally consistent daily series of variables. The baseline climate used to calibrate weather generator parameters, including variable means, standard deviations and inter-variable relationships, uses historic 5 km gridded data for the period 1960 to 1996 interpolated from the UK weather station network using elevation, eastings/northings, and distance from coast [46–48]. Weather sequences under future climate conditions are generated by perturbing parameters using monthly change factors drawn from the probability distribution functions and expressed as the difference (or ratio) between baseline and future scenarios.

For the purposes of this study, one thousand 30-year daily weather sequences were generated for the projected climate conditions for each of three thirty-year time periods (2010–39, 2040–69, 2070–99) under low, medium and high emissions scenarios that respectively correspond to A1FI, A1B and B1 IPCC SRES scenarios [49]. Each simulation also produced 1,000 30-year weather sequences under baseline climate conditions (1961–1990). Weather sequences were generated for a 5 x 5 km square grid cell (UKCP09 cell ID 2050070), corresponding to the location of an established commercial vineyard in Cornwall. Historical daily maximum and minimum temperature data corresponding to the same 5 x 5 km grid cell was acquired for the years 1960 to 2011.

### Grapevine phenology models

The most widely used grapevine phenological models are spring warming or growing degree day (GDD) models, which determine the timing of yearly phenophases, such as budbreak, flowering and veraison (when grapes begin to soften and change colour), by the date at which an accumulated measure of daily temperature (or ‘forcing’) attains a critical value [50–52]. A non-linear effect of temperature is described by some models using a sigmoidal response curve or by incorporating upper or lower temperature thresholds [53]. Other models have sought to simulate the timing of multiple phenological stages [54], the effect of a chilling requirement (vernalization) on budbreak [55, 56], or form part of more complex crop growth models [57]. Model parameters are generally calibrated for different grapevine cultivars and cultivation sites, but for many marginal or newly developing viticulture regions such calibration is not possible due to a lack of empirical data. Comparisons of different grapevine phenological models [12, 58–60] have tended to find simple GDD models to be equal or more spatially and temporally robust than more complex models. However, vernalization may play a greater role in determining phenology under future climate change scenarios [61].

For the purpose of this study we apply two sets of models from the literature using parameters for the Chardonnay cultivar which is currently the most widely planted grapevine in England [20]. The first set of models comprises simple GDD budbreak [58], flowering and veraison models [60, 62]. The models of flowering and veraison were developed from a wider range of cultivars and range of locations across northern, central and southern Europe than any comparable phenology model; no such extensively calibrated budburst model exists. We refer hereafter to these as ‘spring warming’ models for which the temperature forcing equation and parameters are described in Table 1.

**Table 1:** Parameters & forcing equation for spring warming models [58, 60, 62] used to simulate the timing of budbreak, flowering and veraison. The same forcing equation, using different starting times and base temperatures and where *T*_*mean*_ is the mean temperature of day *t*, applies to all three phenophases.

	Budbreak	Flowering	Veraison
Start day (t_0_) of forcing.	1	60	60
Forcing state (FS) on day t	$\sum_{t0}^{t}\text{max}\left[\left(T_{\bar{mean}}-T_{base}\right),0\right]$		
T_base_ used to calculate FS	5°C	0°C	0°C
FS at which phenophase occurs.	318	1217	2547

The second set of linked models [53] incorporate a winter chilling (or vernalization) requirement that determines the time of dormancy break from which temperature forcing of budbreak begins. These models derived from a tree budburst model [55] and were developed and validated for several viticulture regions of northern Italy with parameter values informed by grapevine biology and experimental studies. The relationship between the accumulation of chilling and temperature forcing units to daily mean temperatures are described by bell-shape and sigmoidal functions respectively, and the starting point of accumulation is determined by the timing of the previous phenophase. Given the extended growing season found in Cornwall, the time at which chilling units begin to be accumulated was set to the 1^st^ October. We refer to these as ‘winter chilling’ models and the forcing equations and parameters are given in Table 2.

**Table 2:** Parameters and equations of winter-chilling models [53] used to simulate the timing of dormancy break, budbreak and flowering. Daily chilling and forcing states calculated from daily mean temperature (T_mean_) and curve shape parameters a = 0.005 and c = 2.8. The same forcing equation applies to budbreak and flowering. The critical forcing state at which budburst occurs is calculated from the chilling state and curve shape parameters: co1 = 176 and co2 = 0.015.

Model parameter	Dormancy break	Budbreak	Flowering
Start day (t_0_) of chilling / forcing.	1^st^ October	Dormancy break	Budbreak
Chilling (CS) or forcing state (FS) on day t	$CS=\sum_{t_{0}}^{t}\frac{2}{{1+e}^{{a(T_{mean}-c)}^{2}}}$	$FS=\sum_{t_{0}}^{t}\frac{1}{{1+e}^{-0.26(T_{mean}-16.06)}}$	
CS or FS at which phenophase occurs.	79	= *co*1 * *e* ^*co*2*CS^	25

### Risk definition and calculation

The risk of frost damage is determined not only by the probability of frost after budbreak, but also the frequency and the severity of frosts determined by the duration and intensity of sub-zero temperatures to which plants are exposed. We calculated three alternative measures of risk: (i) the probability of a late frost defined as the probability of a minimum temperature < = 0°C on any day after budbreak but before flowering; (ii) the frequency of late frost defined as the number of days after budbreak when the minimum temperature is < = 0°C, and (iii) a measure of the severity of late frost defined as the accumulated negative degree days below 2°C after budbreak.

Adverse weather such as precipitation or low temperatures during the flowering period, that typically lasts for about two weeks, can be a cause of poor fruit set. A sigmoidal relationship has been observed between Chardonnay bunch weight and flowering temperatures 13.8 to 19.6°C [63]. For the purpose of this study, we have defined adverse flowering weather as any day with a daily mean temperature less than 15°C or total daily precipitation of over 5 mm, calculating (i) the number of days within 7 days before or after flowering having adverse conditions and (ii) the probability of ten or more such days within 7 days either side of flowering.

Seasonal cumulative growing degree days corresponding to the widely used Winkler index [64], calculated using a base temperature of 10°C from 1^st^ April to end of October, were also determined for each yearly weather sequence.

Growing degree days and the risk of spring frost and adverse flowering weather were determined for each year of each weather sequence generated, using budbreak and flowering dates modelled from the weather for that same year. Risk measures were expressed as a mean value or a probability of occurrence under each emissions scenario and time period.

The probabilities of a latefrost and of adverse flowering weather were also calculated as a function of the day of year, allowing the generation of mean seasonal risk profiles under different climate scenarios. The calculation of such profiles revealed a distinct, monthly stepwise pattern to their variation that was most clearly evident when profiles of mean daily precipitation and mean temperature were calculated (S1 Fig). This monthly pattern is an artefact explained by two aspects of the UKCP09 weather generator. Firstly weather series under future climatic conditions are generated by the application of monthly change factors derived from the climate models, and secondly seasonal calibration of the weather generator was based upon a comparison of generated and observed statistics for half-monthly time periods [7]. As a result, larger changes in weather are more likely to be generated across monthly (and half-monthly) boundaries than within months. This monthly pattern does not remain constant under different climate scenarios, with profiles for later time periods and higher emissions scenarios showing much higher transitions between certain months, reflecting the greater seasonal variation in precipitation (namely higher winter precipitation and lower summer precipitation) under future climate scenarios. To remove the effect of these artefacts, daily risk profiles were re-calculated using rolling 31-day averages.

All modelling and calculations were carried out using iterative routines programmed using R statistical software [65].

## Results

We describe firstly variation in seasonal growing degree days and the application of spring warming models to simulate grapevine phenology from which the exposure to late frost and adverse flowering weather under different climate scenarios. Subsequently we compare these results with those obtained using winter chilling models to simulate grapevine phenology.

### Risk exposure under different climate scenarios

**Fig 2:**
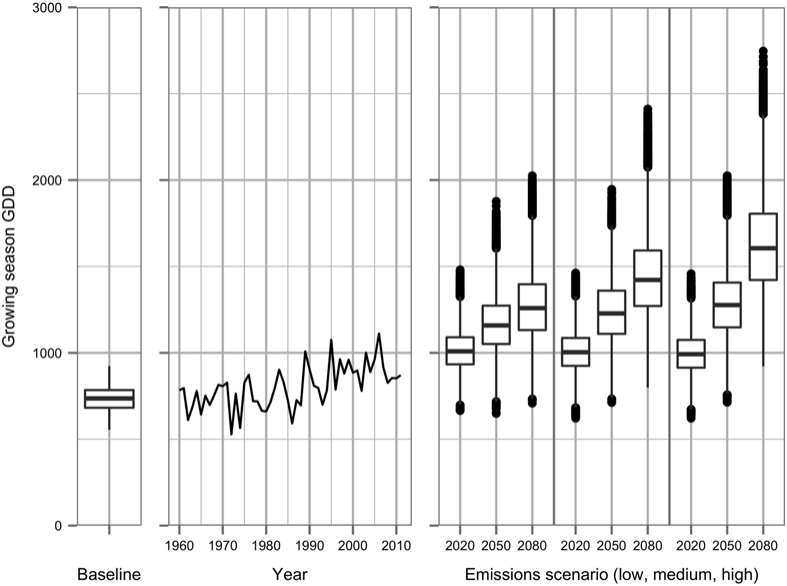
Changes in cumulative growing season GDD (1^st^ April to 31^st^ October) calculated from baseline (1960–90) weather generator runs, daily historic weather data (1960–2011) and future climate weather generator runs using three emissions scenarios (low, middle and high emissions from left to right) and time periods. Baseline quantile box plots calculated from 9,000 30-year time sequences; future box plots from 1,000 30-year time sequences.

The higher temperatures predicted under future climate scenarios and time periods are reflected in projected increases in the seasonal growing degree days measured from 1^st^ April to 31^st^ October, with the historic weather record also showing an increasing trend in seasonal GDD since 1961, despite year-on-year variation (Fig 2). The variation in seasonal GDD represented by box plots for the same climate scenario includes variation from differences between each of the 1,000 future climate projections as well as year-on-year variation within each 30-year sequence.

**Fig 3:**
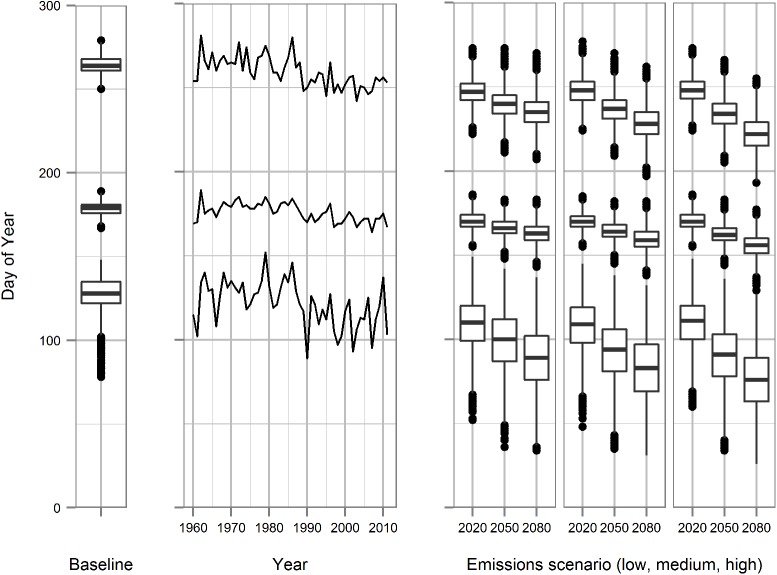
Modelled times of budbreak, flowering and veraison (from bottom to top) calculated from baseline (1960–90) weather generator runs, daily observed weather (1960–2011) and future climate weather generator runs using three emissions scenarios (low, middle and high emissions from left to right) and time periods.

The warmer temperatures under future climate scenarios result in earlier model dates of budbreak, flowering and veraison compared with the baseline climate and the historical weather record from 1961 to 2010 (Fig 3) using spring warming models. From baseline climate conditions, budbreak is predicted to advance by 45 days, and flowering and veraison by 19 days and 37 days respectively under the medium emission scenario by 2080.

Table 3 describes how late frost risk and adverse flowering weather vary between different climate scenarios using spring warming models to describe grapevine phenology. Despite the increase in growing season temperatures, the table indicates an increased late frost risk under many future climate scenarios as a result of the advancement in budbreak.

**Table 3:** Mean seasonal growing degree days, measures of late frost risk and of adverse flowering weather under different climate scenarios. Late frost risk expressed as (i) probability of a frost day (minimum temperature < = 0°C) after budbreak; (ii) mean number of these frost days, and (iii) mean accumulated degree days under 2°C after budbreak. Adverse flowering weather defined as a mean daily temperature <15°C or total precipitation>5mm and expressed as (i) the probability of 10 or more adverse days during the 7 days before and after flowering, and (ii) mean number of adverse days during the same 15 day period. The timing of budbreak and flowering calculated using spring warming models.

Emissionsscenario	Time period	Seasonal GDD	Frost risk measures			Adverse flowering weather	
			Probability of frost	Number of frost days	Degree days <2°C	Probability of 10+ adverse days	Number of adverse days
Baseline	1961–1990	734	0.03	0.03	0.06	0.39	7.5
	2010–2039	1014	0.16	0.11	0.40	0.13	4.7
Low	2040–2069	1167	0.23	0.16	0.59	0.11	4.0
	2070–2099	1271	0.23	0.16	0.59	0.11	3.9
	2010–2039	1008	0.20	0.13	0.50	0.14	4.7
Medium	2040–2069	1240	0.24	0.16	0.61	0.10	3.7
	2070–2099	1444	0.22	0.15	0.57	0.09	3.1
	2010–2039	997	0.17	0.12	0.44	0.17	5.1
High	2040–2069	1283	0.23	0.16	0.60	0.11	3.7
	2070–2099	1628	0.20	0.13	0.52	0.08	3.0

The different measures used to describe the risk of late frost damage show similar variation between different climate scenarios in Table 3, and we henceforth adopt the probability of one or more days of frost after budbreak as a measure of ‘late frost risk’, while noting that near identical results are found using measures that capture aspects of the severity or frequency of frost events.

**Fig 4:**
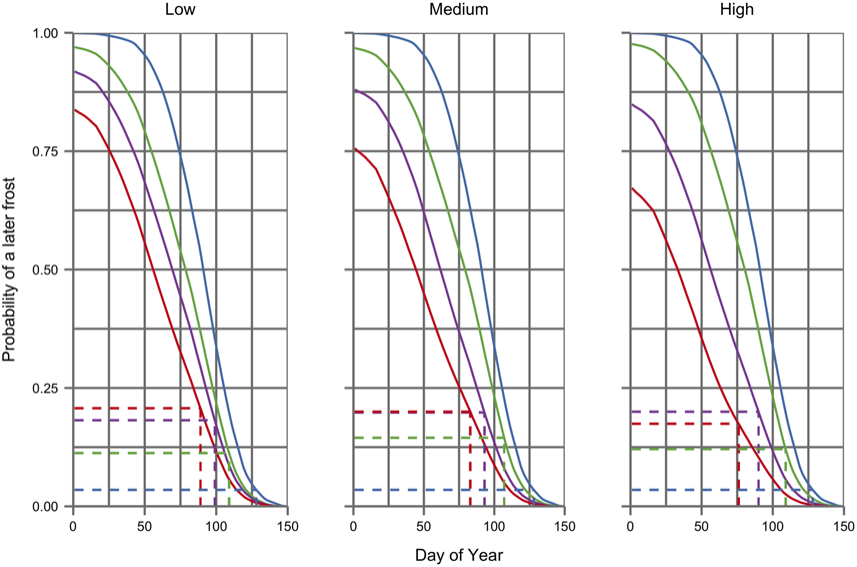
Spring frost risk profiles under baseline (blue) and three future time periods (green 2010–39, purple 2040–69, red 2070–89) for low, medium and high emissions scenarios (from left to right). Dotted lines indicate the mean budbreak date, calculated using a spring warming model [58], and the corresponding risk of a later frost under each scenario and time period.

**Fig 5:**
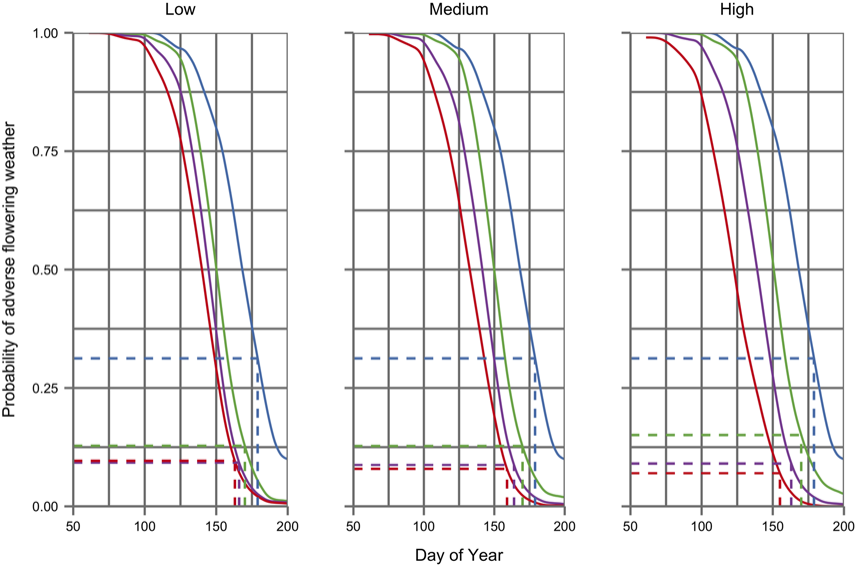
Risk profiles of adverse flowering weather (defined as 10 or more of the 14 days after flowering with daily mean temperatures <15°C or >5mm precipitation) under baseline (blue) and three future time periods (green 2010–39, purple 2040–69, red 2070–89) for low, medium and high emissions scenarios (from left to right). Dotted lines indicate the mean budbreak date, calculated using a spring warming models [60, 62], and the corresponding risk of adverse flowering weather under each scenario and time period.

Fig 4 illustrates the probability of frost under different climate scenarios as a function of the day of the year. Under future climate scenarios the sigmoidal frost risk profile shifts to the left and downwards as the probability of frost declines and they occur earlier in the season. Yet despite this shift in the frost risk profile, the figure confirms how an advancement in mean budbreak times under future scenarios (illustrated with their corresponding frost risk by dotted lines in Fig 4) is sufficient to increase the risk of late frost. In contrast, Fig 5 shows how the smaller phenological shift in the time of flowering has little effect on exposure to adverse flowering weather, which declines from a frequency of over 1 in 3 years during baseline years to about 1 in 10 under many future climate scenarios (see also Table 3). The risks indicated in Figs 4 and 5, calculated from mean phenological timings and mean daily risks under each scenario, differ slightly from those in Table 3 where the risk of frost or adverse flowering weather is calculated for each individual year using the dates of budbreak or flowering for that year.

### Risk exposure using different phenological models

The use of winter chilling models [53] produces very different trends in budbreak times and frost risk exposure under future climate projections than those calculated from simpler spring warming models.

The timing of budbreak varies much less between different climate scenarios when using winter chilling models. Although an advancement of 7 days from baseline conditions is predicted under the 2070–99 medium emissions scenario (Table 4), this is much less than that predicted using the spring warming models. In effect the warmer winter conditions of future climate projections delay the timing of dormancy break, thereby compensating for the increased budbreak forcing due to warmer spring weather. As a result of the smaller advancement in budbreak times, late frost risk declines under all future scenarios. In contrast, the times of flowering resemble those predicted using spring warming models and provide similar estimates of exposure to adverse flowering weather under future conditions.

**Table 4:** Mean budbreak and flowering times under different climate conditions using two types of phenology model: (i) spring warming budbreak and flowering models [58, 60, 62], and (ii) winter chilling (vernalization) models [53].

Emissionsscenario	Time period	(i) Spring warming models		(ii) Winter chilling model	
		Budbreak	Flowering	Budbreak	Flowering
Baseline	1961–1990	128	179	113	192
	2010–2039	109	170	110	181
Low	2040–2069	99	166	108	176
	2070–2099	89	163	106	172
	2010–2039	107	170	109	181
Medium	2040–2069	93	164	107	173
	2070–2099	83	159	106	168
	2010–2039	109	170	110	182
High	2040–2069	90	163	106	171
	2070–2099	76	155	106	163

## Discussion

This study demonstrates the application of a simple ‘agro-climate ensemble model’ [66] to capture the effects of climate change on agricultural risks associated with key phenological events that are also affected by seasonal conditions in terms of their timing. Despite improvements to average conditions for wine growing in cool-climate regions, our study demonstrates how exposure to adverse conditions at key stages in crop development is less readily predicted.

The projected increase in growing season temperatures under future climate scenarios, from under 800 GDD to over 1,200 GDD even under the low emissions scenario, suggests cultivation of a much wider range of cultivars and more reliable and higher quality harvests of wine grapes will be possible in the focal cool-climate region under future conditions. Warmer sites in south-east England currently receive about 850 GDD over a typical growing season which is adequate to permit production of high quality sparkling and aromatic white wines, but a GDD of about 1,100 is considered necessary in order to permit high quality, reliable harvests of cultivars such as Chardonnay or Pinot Noir [22].

Of equal importance, the results also indicate that improved flowering conditions and therefore more reliable fruit set and harvest yields, are likely under future climate conditions. However the results advise caution on adopting grapevine varieties more susceptible to frost damage or early budbreak varieties [67], as an advancement in phenology can potentially increase exposure to frost risk.

Several recent studies [29, 61] have reported contrasting frost risk projections under future climate scenarios for different viticulture regions. Such results reflect how the effect of climate change on frost exposure depends on grapevine phenology and the seasonal weather patterns that define the risk profile under present and future climates: factors that vary between different grapevine cultivars and viticulture regions. Figs 3 and 4 provide an indication of how a shift in phenology can increase exposure to what would otherwise be a lowered risk under future climate conditions.

The results of this study, however, indicate that estimates of future crop risk are sensitive not only to future climate projections but also to the choice of phenological model.

Climate models are often seen as the primary source of uncertainty concerning the impacts of future climate change. The UKCP09 projections seek to capture much of the inherent uncertainty associated with climate models within their probability projections, although their ability to do so and the reliability of their quantified predictions remains contested [68, 69]. The downscaling of future projections to generate weather sequences at high levels of temporal and spatial resolution does not in itself add anything to the reliability of climate projections, and it is essential to recognize that the resulting weather risks are sensitive to their methods of calculation and to changes in the underlying climate probability projections [70].

A greater source of uncertainty for the crop risks we have studied, however, is the choice and reliability of phenology models. Although simple spring warming models have generally been found to be more robust than more complex phenology models when applied across different viticulture regions [62], their applicability is uncertain under conditions where winter chilling requirements are not routinely met [71] as might be the case under future warming scenarios. Experimental studies of the effect of temperature on grapevine phenology also suggest that models derived from long-term phenological records may over-estimate the temperature sensitivity of grapevines [72].

The changes to phenology under future climate conditions reported here [12] using the spring warming models to predict times of flowering [60, 62] and budbreak [58] for the Chardonnay cultivar lie just outside of the range of previous results attained using several different phenological models under future climate change scenarios [12]: budbreak advancement is marginally higher and the shift in flowering slightly less in this study. [72]. Very different risk projections result from the application of the phenology models that incorporate a winter chilling requirement [53] but it is not possible to conclude whether this model more accurately reflects the processes that will affect phenology under future climate conditions. The low spatial and temporal robustness of models reflects a lack of direct correspondence with the biological processes regulating plant response to temperature [12, 73] and our still limited understanding of the mechanisms affecting grapevine phenology, including the degree of phenotypic acclimatization. An additional limitation of the phenology models used, which may have significant implications for their application to future climate scenarios, is their inability to capture the effect on plant growth and development of the projected increase in CO_2_ concentrations: likely to be significant for C_3_ crops such as the grapevine [74].

The provision of relevant and accessible information on clearly defined crops risks can improve the capacity of agriculture to manage the risks, and exploit the opportunities, that will result from future climate change. Such information has particular value to the development of marginal or novel crops, where a lack of cultivation experience and empirical information can heighten risk exposure and hinder appropriate decision-making. By expressing the impacts of climate change by their effect on specific crop risks, the approach demonstrated in this study permits the implications of climate change not only to be readily communicated to vineyard owners and managers, but also to foster knowledge exchange between researchers and practitioners. Dialogue is important to identify how climate change will affect those risks of most importance to regional agriculture. The majority of studies of climate change impacts on viticulture have focussed on changes to growing season temperatures, but those interviewed for this study explained how they could adapt to low growing season temperatures by the selection of appropriate cultivars and wine styles, whereas few options were available for protecting from adverse weather at flowering, which was more determinant of a successful harvest. In effect, although average or accumulated growing season temperatures may define geographical limits to cool-climate viticulture, it does not follow that they are the most important factors determining year-on-year harvest yields or quality in marginal regions.

The lack of robust phenological models is therefore a major limitation to assessing future crop risks and the impacts of climate change on the development of cool-climate viticulture in historically marginal climatic regions

## Supporting Information

S1 Fig Mean daily total precipitation and mean temperatures: calculated from 1,000 thirty-year UKCP09 simulations–example under a Medium emissions scenario under baseline (––) and three future time periods (–– 2010–39,–– 2040–69,–– 2070–89). (TIFF)
