# Supplementary figures and images for: Climate Change and Crop Exposure to Adverse Weather: Changes to Frost Risk and Grapevine Flowering Conditions
Source: PLoS One. 2015 Oct 23;10(10):e0141218. doi: 10.1371/journal.pone.0141218 (PMC4619710; doi:10.1371/journal.pone.0141218)

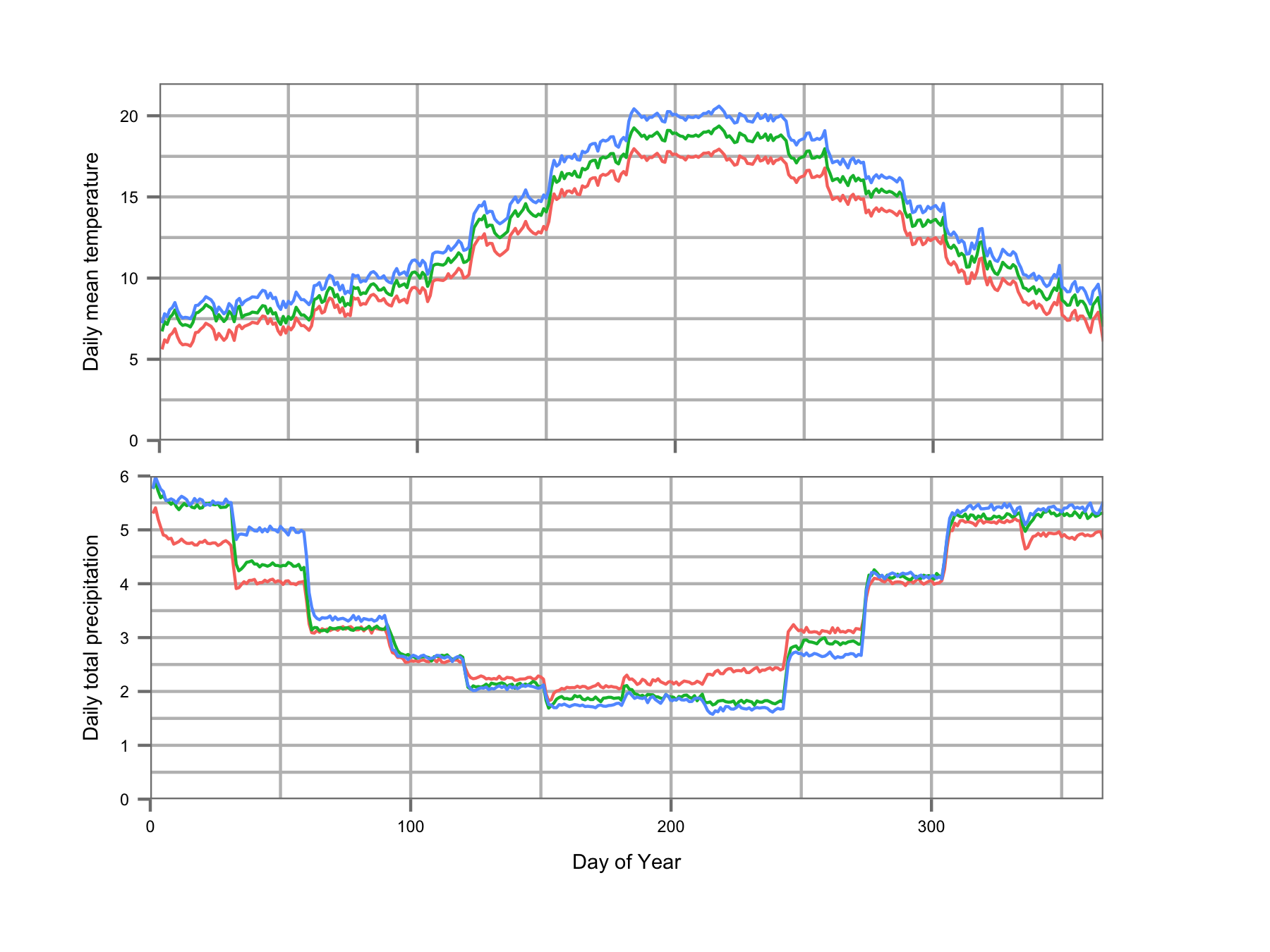

Supplement: S1 Fig — (TIFF) [file pone.0141218.s001.tiff]
